# Differences in risk factors of malignancy between men and women with type 2 diabetes: A retrospective case-control study

**DOI:** 10.18632/oncotarget.17716

**Published:** 2017-05-09

**Authors:** Mariusz Dąbrowski, Elektra Szymańska-Garbacz, Zofia Miszczyszyn, Tadeusz Dereziński, Leszek Czupryniak

**Affiliations:** ^1^ University of Rzeszow, Faculty of Medicine, Institute of Nursing and Health Sciences, Rzeszów, Poland; ^2^ Medical University of Łódź, Department of Infectious and Liver Diseases, Łódź, Poland; ^3^ Private Clinic of Internal Diseases and Diabetes, Przemyśl, Poland; ^4^ NZOZ Esculap, Gniewkowo, Poland; ^5^ Warsaw Medical University, Department of Internal Diseases and Diabetology, Warsaw, Poland

**Keywords:** cancer, diabetes, gender, insulin, metformin

## Abstract

**Background:**

The aim of this multicenter, retrospective, case-control study was to identify differences in risk factors of malignancy between men and women with type 2 diabetes.

**Results:**

Among women the most prevalent malignancies were: breast and uterine cancers (35.6% and 14.4% respectively), while among men there were: colorectal and prostate cancers (24.5% and 13.3% respectively). In both gender metformin use was associated with lower cancer risk. Obesity and insulin treatment in dose-dependent and time-varying manner were associated with significantly increased risk of malignancy in females. In men, unexpectedly, cardiovascular disease was more prevalent in control group. Other variables did not show significant association with malignancy risk.

**Materials and Methods:**

118 women and 98 men with type 2 diabetes mellitus who developed cancer after diagnosis of diabetes and the same number of strictly age matched controls with type 2 diabetes and without malignancy were included into the study. Diabetes duration, antidiabetic medications use, glycated hemoglobin level, body mass index, smoking habits, occupation, presence of comorbidities and aspirin use were included into analyses.

**Conclusions:**

Metformin demonstrated protective effect against cancer in both sexes. Obesity and insulin treatment seem to have greater impact on cancer risk among women.

## INTRODUCTION

The interest of scientific world in the relationship between diabetes and cancer has a long history [1, 2]. Meta-analyses published in the last decade documented that several types of malignant neoplasms are significantly more prevalent among subjects with type 2 diabetes mellitus (T2DM) compared to general population [3–11]. Numerous factors, both genetic and environmental, are involved in cancer development and progression [12–13]. Among people with T2DM obesity, insulin resistance, hyperinsulinemia, hyperglycemia, and inflammation seem to play key roles in elevating cancer risk [14–16].

Antidiabetic medications have been long assumed to be able to modulate risk of malignancy. There is increasing evidence indicating protective role of metformin in cancer development and outcomes, which was summarized in several meta-analyses [17–20]. Conversely, data from observational studies indicate an increased cancer risk associated with the use of exogenous insulin [20–22]. The use of sulfonylurea (SU) derivatives, still one of the most frequently used oral antidiabetic medications, also seem to be associated with the risk of malignancy, however, in one of the meta-analyses of case-control studies these drugs demonstrated neutral impact on cancer risk [19, 20]. Upon existing evidence, oncogenic effect of other antidiabetic medications on several site-specific cancers cannot be neither completely excluded nor clearly confirmed, and its impact on cancer risk still remains a matter of uncertainty [20, 23].

Since in the vast majority of published studies risk of malignancy in T2DM has not been analyzed separately for each gender, it is therefore difficult to assess to what extent risk factors of cancer in this population are gender related. Thus, the main objective of the continuation of our multicenter, retrospective, case-control study [24] was to assess whether women and men with T2DM have similar or different risk factors of cancer development.

## RESULTS AND DISCUSSION

**Figure 1:**
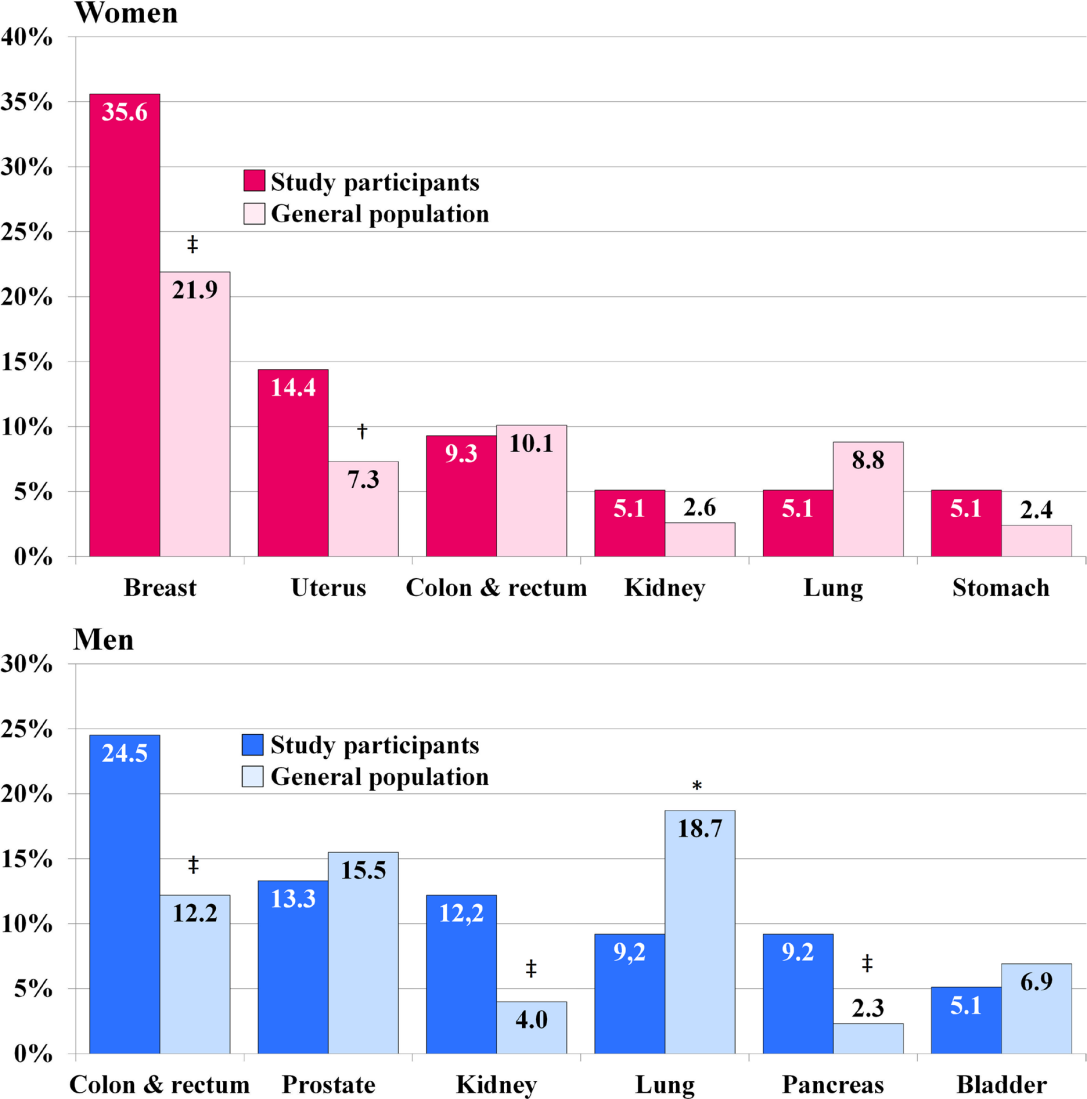
Distribution of site-specific cancers among study participants with malignancy compared to its distribution in the whole Polish population in the year 2013 [25] * *P* < 0.05, ^†^*P* < 0.01, ^‡^*P* < 0.001.

Characteristics of case and control groups separately for each gender is presented in Table 1. Mean age at the time of cancer diagnosis (index time) was not significantly different between women and men. Age distribution (< 45, 45–54, 55–64, 65–74 and ≥ 75 years) was also not significantly different between males and females with cancer. 75 women (63.6%) and 54 men (55.1%) were aged ≥ 65 years. The distribution of the most common cancer sites among the study participants in relation to its distribution in the whole Polish population in 2013 [25] is shown in the Figure 1 (proportions of site-specific cancers in Poland were rather stable in the last decade). Despite relatively small number of cases, proportion of breast and uterine cancers among women and colorectal, kidney and pancreatic cancers among men was significantly higher compared to general population, while proportion of lung cancer among men was lower.

**Table 1:** Characteristics of case and control groups with regard to gender

Parameter	Women			Men			Women vs. Men
	Cancer	Control	*P* value	Cancer	Control	*P* value	*P* value
*n*	118	118	N/A	98	98	N/A	N/A
Age at index time (years)	67.8 ± 9.8	67.8 ± 9.8	N/A	66.3 ± 9.6	66.3 ± 9.7	N/A	N/S
Diabetes duration (years)	11.1 ± 7.8	10.8 ± 8.3	N/S	10.3 ± 7.0	10.3 ± 7.8	N/S	N/S
HbA_1c_ (%)	7.37 ± 1.21	7.36 ± 1.05	N/S	7.34 ± 1.16	7.23 ± 1.07	N/S	N/S
Diabetes treatment (%)							
Metformin	**61.9%**	**80.5%**	**0.003**	**66.3%**	**83.7%**	**0.008**	N/S
Sulfonylurea derivatives	36.4%	N/S	48.3%	N/S	52.0%	N/S	N/S
Acarbose	6.8%	5.9%	N/S	48.0%	10.2%	7.1%	N/S
DPP-4 inhibitors	**1.7%**	2.5%	N/S	12.2%	7.1%	N/S	**0.002**
Insulin	**56.8%**	41.5%	0.027	48.0%	39.8%	N/S	N/S
Insulin therapy duration (years)	**7.4 ± 6.3**	6.5 ± 4.5	N/S	4.6 ± 3.6	7.0 ± 5.6	N/S	**0.011**
Insulin dose (IU/kg)	**0.64 ± 0.31**	0.56 ± 0.24	N/S	0.51 ± 0.25	0.47 ± 0.22	N/S	**0.034**
BMI (kg/m^2^)	**31.7 ± 5.7**	30.1 ± 5.0	0.017	29.6 ± 4.5	30.2 ± 4.4	N/S	**0.009**
Occupation (%)							
Rural	22.9%	22.0%	N/S	16.3%	21.4%	N/S	N/S
Town < 50.000 inhabitants	12.7%	19.5%	N/S	13.3%	14.3%	N/S	N/S
City > 50.000 inhabitants	64.4%	58.5%	N/S	70.4%	64.3%	N/S	N/S
Smoking habits (%)							
Never smokers	**77.8%**	72.0%	N/S	**29.6%**	38.8%	N/S	**< 0.001**
Past smokers	**14.5%**	20.3%	N/S	**39.8%**	38.8%	N/S	**< 0.001**
Current smokers	**7.7%**	7.7%	N/S	**30.6%**	22.4%	N/S	**< 0.001**
Comorbidities (%)							
Hypertension	90.7%	89.0%	N/S	83.7%	87.8%	N/S	N/S
Hyperlipidemia	78.8%	78.8%	N/S	74.5%	80.6%	N/S	N/S
Cardiovascular disease	25.4%	17.8%	N/S	**24.5%**	**38.8%**	**0.046**	N/S
Aspirin use (%)	56.6%	48.6%	N/S	**46.8%**	**61.1%**	**0.0499**	N/S

Data are presented as number, mean ± standard deviation (SD) or proportion (significant differences in bold).

Abbreviations: N/A, non-applicable; N/S, non-significant.

### Metabolic control

**Figure 2:**
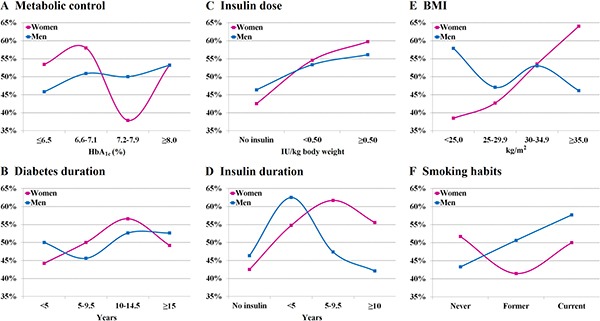
Proportion of patients with malignancy among women and men across categories of analyzed variables

Mean HbA_1c_ level was not significantly different between the case and control groups, regardless of gender (Table 1). Also when patients were divided into four quartiles according to HbA_1c_ values, no significant differences between cancer and control groups were found (Table 2). Among men the lowest risk of malignancy was observed among patients with HbA_1c_ level ≤ 6.5%, while among women it was revealed at the range between 7.2% and 7.9% (Figure 2A). Male subjects with HbA_1c_ value ≥ 8.5% had elevated risk of malignancy compared to patients with lower HbA_1c_ values, OR 2.36 (0.97–5.76), but it did not attain statistical significance, *P* = 0.054. Among women this relationship was even less pronounced. Overall impact of glycated hemoglobin on cancer risk was discussed in details elsewhere [24]. In a recently published meta-analysis increasing HbA_1c_ levels were related to elevated risk of colorectal, pancreatic, respiratory and female genital tract cancers, while they were not related to risk of breast, gastrointestinal or urological cancers [26]. In our study colorectal, pancreatic, respiratory and uterine cancers were present in 32.2% of women and in 42.9% of men. Females with these malignancies had significantly higher HbA_1c_ level compared to women with other cancer sites (7.65 ± 1.20% vs. 7.23 ± 1.19% respectively, *p* = 0.048). In males this difference, although apparent (HbA_1c_ 7.53 ± 1.30% vs. 7.10 ± 0.98% respectively), it did not attain statistical significance. In a rare study analyzing HbA_1c_ level and cancer risk separately for men and women no association was found among male subjects, while in female patients relationship was significant, but non-linear [27].

**Table 2:** Odds ratios (OR) and, in parentheses, 95% confidence interval (CI) associated with analyzed variables (significant differences in bold)

Parameter	Women		Men	
	OR (95% CI)	*P* value	OR (95% CI)	*P* value
Diabetes duration				
< 5 years	Ref.	N/A	Ref.	N/A
5.0–9.9 years	1.26 (0.61–2.60)	N/S	0.84 (0.38–1.84)	N/S
10–14.9 years	1.65 (0.76–3.56)	N/S	1.11 (0.51–2.44)	N/S
≥ 15 years	1.21 (0.58–2.55)	N/S	1.11 (0.47–2.65)	N/S
HbA_1c_				
≤ 6.5%	Ref.	N/A	Ref.	N/A
6.6–7.1%	1.20 (0.56–2.58)	N/S	1.22 (0.57–2.64)	N/S
7.2–7.9%	0.53 (0.26–1.09)	N/S	1.18 (0.52–2.68)	N/S
≥ 8.0%	0.99 (0.48–2.03)	N/S	1.34 (0.60–3.01)	N/S
Antidiabetic medications (use vs. non-use)				
Metformin	**0.39 (0.22–0.71)**	**0.003**	**0.38 (0.20–0.76)**	**0.008**
Sulfonylurea derivatives	0.61 (0.37–1.03)	N/S	0.85 (0.49–1.49)	N/S
Insulin	**1.85 (1.10–3.10)**	**0.027**	1.39 (0.79–2.46)	N/S
Insulin therapy duration				
No insulin	Ref.	N/A	Ref.	N/A
< 5 years	1.64 (0.81–3.32)	N/S	1.93 (0.96–3.80)	0.062
5–9.9 years	**2.18 (1.09–4.35)**	**0.039**	1.04 (0.39–2.76)	N/S
≥ 10 years	1.69 (0.73–3.92)	0.84 (0.31–2.25)	N/S	N/S
Insulin dose				
No insulin	Ref.		Ref.	
< 0.50 IU/kg	1.62 (0.81–3.25)	N/S	1.32 (0.66–2.65)	N/S
≥ 0.50 IU/kg	**2.01 (1.11–3.63)**	**0.031**	1.48 (0.72–3.04)	N/S
BMI				
< 25.0 kg/m^2^	Ref.	N/A	Ref.	N/A
25.0–29.9 kg/m^2^	1.19 (0.49–2.92)	N/S	0.65 (0.24–1.77)	N/S
30.0–34.9 kg/m^2^	1.84 (0.74–4.61)	N/S	0.82 (0.29–2.30)	N/S
≥ 35.0 kg/m^2^	2.84 (1.07–7.57)	**0.034**	0.62 (0.19–2.06)	N/S
Occupation				
Rural	Ref.	N/A	Ref.	N/A
Town < 50.000 inhabitants	0.63 (0.27–1.46)	N/S	1.23 (0.64–2.37)	N/S
City > 50.000 inhabitants	1.06 (0.57–1.99)	N/S	1.63 (0.78–3.39)	N/S
Smoking habits				
Never smokers	Ref.	N/A	Ref.	N/A
Past smokers	0.66 (0.33–1.32)	N/S	1.35 (0.70–2.60)	N/S
Current smokers	0.93 (0.35–2.46)	N/S	1.79 (0.86–3.72)	N/S
Comorbidities				
Hypertension	1.20 (0.52–2.81)	N/S	0.72 (0.32–1.60)	N/S
Hyperlipidemia	1.00 (0.54–1.87)	N/S	0.70 (0.36–1.38)	N/S
Cardiovascular disease	1.58 (0.84–2.95)	N/S	**0.51 (0.28–0.96)**	**0.046**
Aspirin use	1.38 (0.82–2.33)	N/S	**0.56 (0.32–1.00)**	**0.0499**

Abbreviations: N/A, non-applicable; N/S, non-significant.

Although increased risk of malignancy was found also in higher versus lower values of HbA_1c_ within a normal range [27], it can be more noticeable at a diabetic range. Glucose is a primary energy source for cancer cells, and thus higher glucose concentrations may accelerate cancer growth and increase it invasiveness [28–30]. Prolonged hyperglycemia leads to enhanced formation of reactive oxygen species (ROS) and to accumulation of advanced glycation end products (AGEs) which through binding to its specific receptor RAGE (receptor for AGE) lead to activation of the nuclear transcription factor NF-κB and intra-nuclear formation of ROS, which in turn causes DNA damage and exerts mutagenic effect. This pathway can play important role in carcinogenesis [31, 32]. It can explain apparent increase of cancer risk observed in our study among men at the level of HbA_1c_ ≥ 8.5%. Statistical significance was not reached, likely due to a small number of patients with such poor metabolic control. However, not only prolonged hyperglycemia, but also acute glucose level fluctuations, frequently seen in diabetic patients, can lead to increased ROS formation [33].

### Diabetes duration

Duration of diabetes did not differ significantly between patients with and without malignancy, both among women and men (Table 1). Also when patients were divided into four subgroups according to diabetes duration, no significant differences were revealed neither in female nor in male population (Table 2, Figure 2B).

Effect of diabetes duration on the risk of cancer remains a matter of controversy. In two large, registry-based, retrospective cohort studies Johnson et al. and Carstensen et al. independently found the highest number of diagnosed cancer early after diabetes onset, both among women and men [34, 35]. This phenomenon was explained by Johnson et al. by increased ascertainment at the time of diabetes diagnosis [34]. Opposite results were obtained in other large cohort study by Li et al., in which the lowest risk of malignancy has been observed in the first 5 years from the onset of diabetes, while the highest cancer risk among patients suffering from diabetes for over 15 years. Also in this study gender did not modulate this risk [36]. The increasing risk of cancer incidence with a longer duration of diabetes observed in this study was explained by cumulative effect of hyperglycemia, use of insulin and weight gain during the course of the disease. In addition, the fact that in some cases cancer may be un underlying cause of diabetes (cf. pancreatic cancer) is also a confounder in the analysis of the effect of diabetes duration on cancer risk. Older age is also strongly associated with increasing cancer risk both in diabetic and non-diabetic population, regardless of gender [25, 37]. In our study the lowest risk of malignancy among females was observed in the first 5 years, while the highest risk between 10 and 15 years after diabetes onset. However, these differences were insignificant. Among men cancer occurrence was stable throughout the whole course of diabetes (Figure 2B).

### Diabetes treatment

Significantly fewer patients in the case groups were treated with metformin compared to the control groups in both sexes (Tables 1 and 2). This difference remained significant also in all models of the multiple logistic regression analysis (Table 3). Among patients treated with metformin mean duration of this therapy was not significantly different between case and control groups, both among women (10.4 ± 7.2 vs. 10.6 ± 8.2 years respectively) and among men (10.3 ± 6.4 vs. 10.3 ± 8.1 years respectively). The only significant difference was higher mean BMI in women who developed cancer compared to those without malignancy, 33.0 ± 5.8 vs. 30.3 ± 4.8 kg/m^2^ respectively, *P* = 0.002. Among men such difference was not observed (BMI 30.6 ± 4.6 vs. 30.5 ± 4.5 kg/m^2^ respectively).

**Table 3:** Antidiabetic medications and malignancy risk in the multiple logistic regression analysis (significant differences in bold)

Antidiabetic medication	Women		Men	
	OR (95% CI)	*P* value	OR (95% CI)	*P* value
Metformin				
Model 1	**0,42 (0,23–0,78)**	**0.006**	**0,38 (0,19–0,77)**	**0.007**
Model 2	**0.38 (0.20–0.72)**	**0.003**	**0.33 (0.15–0.70)**	**0.004**
Model 3	**0.39 (0.21–0.75)**	**0.005**	**0.34 (0.15–0.75)**	**0.008**
Sulfonylurea derivatives				
Model 1	0,69 (0,39–1,23)	N/S	0,88 (0,47–1,64)	N/S
Model 2	0,72 (0,40–1,31)	N/S	0,95 (0,50–1,82)	N/S
Model 3	0,74 (0,41–1,35)	N/S	1,00 (0,51–1,98)	N/S
Insulin				
Model 1	1,32 (0,74–2,37)	N/S	1,23 (0,65–2,33)	N/S
Model 2	1,58 (0,78–3,21)	N/S	1,34 (0,63–2,82)	N/S
Model 3	1,57 (0,77–3,21)	N/S	1,60 (0,73–3,51)	N/S

Model associations: Model 1, adjusted to other antidiabetic medications; Model 2, adjusted to antidiabetic medications + HbA1c, diabetes duration and obesity; Model 3, adjusted to all above + smoking habits, occupation and comorbidities.

Abbreviations: N/S, non-significant.

In the univariate analysis insulin therapy was associated with increased risk of malignancy, but this association was significant only in women. In the multiple logistic regression analysis the risk of cancer related to insulin use decreased to a non-significant level (Table 3).

Mean insulin dose and mean duration of insulin treatment did not differ significantly between the case and control groups, regardless of gender (Table 1). However, the risk of cancer was increasing along with insulin dose, and women using insulin at the dose ≥ 0.50 IU/kg had significant, two-fold higher risk of cancer compared to non-users. This relationship was weaker in male patients and did not attain statistical significance (Table 2 and Figure 2C). In women the highest risk of malignancy was observed between 5th and 10th year of insulin therapy, and it was significantly higher compared to non-insulin users, while in men it was found in the first 5 years of insulin treatment, but it was borderline statistically insignificant (*P* = 0.062; Table 2 and Figure 2D). Overall, women with malignancy were treated with insulin significantly longer and in higher doses compared to men with cancer (Table 1). Among patients treated with insulin, men with malignancy were using metformin significantly less frequent compared to men without cancer (55.3% vs. 79.5% respectively, *P* = 0.033). Similar tendency was observed also among women (50.7% vs. 69.4% respectively), but in this case difference did not reach statistical significance, *P* = 0.068. With regard to site-specific cancers, treatment with insulin was significantly more prevalent in the subgroup of men with colorectal cancer in relation to their comparators, 16 vs. 8 patients respectively, *P* = 0.043, and among women with aggregated breast and uterine cancers vs. their comparators, 34 vs. 21 subjects respectively, *P* = 0.027.

SU derivatives in our study appeared to have neutral impact on the cancer risk. For DPP-4 (dipeptidyl-peptidase-4) inhibitors and for acarbose (α-glucosidase inhibitor) odds ratios were not calculated due to a small number of patients treated with these medications, 24 (5.6%) and 32 (7.4%) subjects respectively.

Impact of antidiabetic medications on cancer risk has been widely discussed in the last years. T2DM is a progressive disease and requires intensification of treatment over time. Hyperglycemia is the primary driver of these changes, and the final stage of intensification is insulin therapy in different regimens, with or without concomitant oral or injectable medications (glucagon-like peptide 1 agonists or pramlintide). Time of progression to the next step of treatment regimen varies among different patients, and thus a clear impact of antidiabetic medications on cancer risk is difficult to determine.

Highly significant reduction of cancer risk among metformin users, consistent among men and women observed in our study is in line with the results of meta-analyses published in recent years, which also demonstrated protective effect of metformin on cancer incidence and mortality [17–20] irrespective of gender [20]. Metformin acts as an anti-tumor medication through stimulation of AMP-activated protein kinase (AMPK) and its regulator liver kinase B1 (LKB1), which inhibits the mammalian target of rapamycin (mTOR) and disrupts the life cycle of cancer cells. Moreover, it also indirectly decreases levels of hormones acting as a growth factors (insulin, insulin-like growth factor-1; IGF-1 and sex hormones) [38].

Current evidence from observational studies indicate possible unfavorable effect of insulin therapy on cancer risk in T2DM [20–22, 36, 39, 40]. In our study insulin use was associated with a dose-dependent and duration-related elevated risk of malignancy. However, it was significant only in women. Holden et al. also found in their study impact of insulin dose on increased cancer risk, but inversely, it was significant only in male subjects [39]. The risk of malignancy related to the duration of insulin treatment was in our observation significantly highest between fifth and tenth year of insulin treatment among women, and remained insignificantly elevated with a longer insulin use. In men it was highest in the first 5 years of insulin therapy (borderline insignificant), and then decreased to the neutral level. This phenomenon can be explained by increased cancer and also coronary heart disease (CHD) mortality observed among diabetic patients treated with insulin [40]. In the meta-analysis of observational studies increased risk of malignancy associated with insulin use was highest after 4 years of insulin treatment [21]. Longer duration of insulin therapy and higher doses of insulin in women compared to men with malignancy observed in our study were not analyzed in any of the cited earlier papers. Concomitant metformin use among insulin-treated patients in our study seemed to attenuate negative impact of insulin on cancer risk. Similar effect was also observed by Currie et al. with regards to cancer mortality in T2DM [41].

Impact of insulin on cancer growth is biologically plausible. Insulin is a potent growth-stimulating hormone, which acts through insulin and IGF-1 receptors [29, 42, 43]. Insulin receptor expression is significantly higher in cancer tissues compared to normal cells [43]. However, in prospective studies in T2DM more intensive treatment, frequently with the use of insulin, was not associated with elevated cancer risk [44]. Outcome Reduction with an Initial Glargine Intervention (ORIGIN) trial showed absolutely neutral effect of insulin glargine on cancer risk [45].

SU use in our study did not show significant relationship with cancer risk regardless of gender. Data from other observations are divergent. Thakkar et al. in their meta-analysis revealed increased cancer risk among SU users in cohort studies, while in case-control studies and randomized controlled trials it was neutral [19]. In a more recent meta-analysis SU were associated with elevated cancer risk in all types of observational studies. However, analysis performed separately for women and men demonstrated a neutral effect in both sexes [20].

The number of patients treated with other antidiabetic medications in our study was too small to determine their relationship with cancer risk.

### BMI

Women with malignancy had significantly higher BMI compared to control group, which was not observed in men. Women with cancer had also significantly higher BMI compared with men with malignancy (Table 1) and prevalence of obesity in these groups was 59.3% and 48.0%, respectively. In women risk of cancer was rising along with the increasing BMI (Figure 2E), and obesity (BMI ≥ 30 kg/m^2^) was associated with significantly elevated risk of malignancy, OR 1.92 (95% CI 1.14–3.21), *P* = 0.019 compared to patients with lower BMI, while in men such relationship was not observed (Table 2). In women, after adjustment for all other variables, relationship between obesity and cancer risk became even stronger, OR 2.18 (1.24–3.82), *P* = 0.007.

Obesity is a well-known factor associated with the risk of several types of cancer [46]. In our study association between obesity and risk of malignancy in diabetic population was confirmed, but only in women. Among factors related to increased cancer risk in obese individuals with diabetes the most important ones are insulin resistance, hyperinsulinemia, hyperglycemia, elevated levels and increased bioavailability of insulin-like growth factor-1 (IGF-1), increased pro-inflammatory adipokines concentration and increased bioavailability of sex hormones. Excess of fat mass is associated with increased aromatase activity in adipose tissue and increased conversion of androgens to estrogens, which can has an effect on growth of hormone-related tumors [47]. This mechanism seem to play more important role among women, which is supported by observation that weight reduction after bariatric surgery was associated with reduced cancer incidence only in females and not in males [48, 49].

### Smoking habits

In patients with malignancy number of current and ex-smokers was significantly higher in men compared to women, while number of never-smokers was higher among women (Table 1), P_trend_< 0.001. In men risk of cancer tended to increase in past and current smokers, but the difference did not attain statistical significance (Table 2 and Figure 2F). In the group of men with lung cancer and their comparators number of current and former smokers was significantly higher (8 vs. 2 patients respectively), *P* = 0.015. Among women significant differences were not found.

Smoking is known to be associated with elevated risk of several site-specific cancers, especially lung cancer [50]. No significant differences in number of never, former and current smokers between case and control groups observed in our study (with the exception of lung cancer among men) are likely to result from small number of cancer cases among study participants.

### Other variables

Overall prevalence of cardiovascular disease in all patients was lower in women than in men. (21.6% vs. 31.6% respectively, *P* = 0.024). Interestingly, CVD was significantly more frequent in male controls (Tables 1 and 2), and this association became even stronger after adjustment to all other variables, OR 0.43 (0.21–0.88), *P* = 0.017.

CVD and cancer share similar risk factors including obesity, diabetes, insulin resistance and inflammation [51]. In the study of over 200,000 patients with diagnosed cancer overall prevalence of CVD was 26.6% compared to 17.3% in age-matched non-cancer controls [52]. In another study prevalence of CVD in cancer patients was 19%, and it was 1.7-fold higher in males compared to females [53]. In our study prevalence of documented CVD among women and men with malignancy was not significantly different (Table 1).

For hypertension and hyperlipidemia, we did not observe any significant association with the risk of malignancy.

In the univariate analysis aspirin use demonstrated borderline significant protective effect on the risk of malignancy in male subjects (Tables 1 and 2). Aspirin use is known to be protective against colorectal, esophageal and gastric cancers [54]. In fact, colorectal cancer was the most frequent malignancy found in men participating in our study. However, we did not find any relationship between aspirin use and colorectal cancer. Moreover, after adjustment to the presence of CVD effect of aspirin became insignificant.

Although occupation was found to have an impact on cancer risk [37, 55], in this study we did not find its significant effect on cancer risk, regardless of gender (Tables 1 and 2).

Relatively small number of patients in each gender group should be acknowledged as the most important limitation of our study, having a strong impact on statistical power of our findings. It did not allow us to demonstrate significant relationship with cancer risk for several other variables for which positive or negative trends were observed. Also, we were unable to include into the analysis newer antidiabetic agents due to its infrequent use by the patients. Despite our best efforts also time-related biases cannot be completely excluded [56].

Our study has also several strengths. One of the most important is its case-control design with strictly matched pairs of patients with malignancy and their comparators without cancer, with each pair taken always from the same center. Moreover, our observations were based on a high-quality data sources using individual patients records with a follow-up period to index-time exceeding 10 years in average, and with extensive covariate information, which allowed us to explore the relationship between several risk factors of malignancy associated with T2DM and cancer development in population usually seen in everyday clinical practice. Ethnic homogeneity can be also considered as a strong point of our study, because it allowed us to exclude any ethnicity-related factors associated with cancer risk.

Despite its limitations, the results of our study indicate that in order to minimize cancer risk in men and women with diabetes metformin therapy should be strongly recommended and it should be introduced and maintained throughout the whole course of the disease in all patients (also after introduction of insulin) as long as the drug is well tolerated and no contraindications occur. In addition, insulin therapy in type 2 diabetic patients should be initiated with caution and, if possible, high doses of insulin should be avoided, which is of special importance in women. At least, in insulin treated patients increased oncological vigilance should be maintained in men in the first 5 years of insulin therapy, and in women between fifth and tenth year of insulin therapy. It is also highly reasonable to make strong efforts for weight reduction, especially in women. And finally, deterioration of metabolic control of diabetes should be avoided and increase of HbA_1c_ level above the threshold of 8.5% should not be allowed, especially in men.

## MATERIALS AND METHODS

Inclusion criteria for “case” group were as follows: cancer diagnosed after diagnosis of type 2 diabetes, at least one HbA_1c_ measurement before or at the time of cancer diagnosis, date of diabetes diagnosis, diabetes treatment, BMI and history of comorbidities available. Compared to our previous publication study group was supplemented by 13 new cases, and finally into this analysis we included 118 women and 98 men with T2DM fulfilling inclusion criteria. The control group consisted of strictly age-matched 118 females and 98 males with T2DM without history of cancer and without known malignancy. Data analysis covered the period from January 1998 to 30 September 2016 (the first and the last eligible patient with cancer). Other details of methodology used in our study were described in our previous publication [24].

Statistical analysis of the data was performed using SigmaPlot for Windows version 12.5 (Systat Software Inc., San Jose, CA, USA), and similarly to our previous study it was performed in 2 stages [24]. In the first stage patients were divided according to gender. Then comparison of patients with and without malignancy within the groups, and also between the groups was made. The continuous data were analyzed using an unpaired two-tailed Student's *t*-test or by a Mann-Whitney rank sum test where appropriate. The categorical data were compared using χ^2^ test. In the second stage patients were divided into subgroups according to BMI (< 25.0, 25.0–29.9, 30.0–34.9 and ≥ 35.0 kg/m^2^), diabetes duration (< 5.0, 5.0–9.9, 10.0–14.9 and ≥ 15.0 years), quartiles of HbA_1c_ level (≤ 6.5%, 6.6–7.1%, 7.2–7.9% and ≥ 8.0%), insulin dose (no insulin, < 0.50 and ≥ 0.50 IU/kg), and duration of insulin treatment (no insulin, < 5.0, 5.0–9.9 and ≥ 10.0 years). In addition analysis of the subgroups of patients treated with metformin and insulin was performed. For the assessment of the effect of treatment or analyzed risk factors on cancer occurrence OR (odds ratios) and 95% CI (confidence intervals) were calculated in univariate and in multiple logistic regression models. For comparison between site-specific cancer distribution in the study group and in the whole Polish population a *z*-test was used. A *P* value < 0.05 was considered statistically significant.

The study was approved by the institutional Bioethics Committee at the University of Rzeszow (Resolution number 13/03/2014, stated on 19th March 2014) and by the all appropriate administrative bodies. The study was conducted in accordance with ethical standards laid down in an appropriate version of the Declaration of Helsinki (as revised in Brazil 2013) and in Polish national regulations. According to Polish regulations in non-interventional, retrospective, epidemiological studies, where patients’ data are analyzed anonymously, informed consent is deemed unnecessary and it was not obtained.

## Abbreviations

AGEs: Advanced glycation end products
AMPK: AMP-activated protein kinase
BMI: Body mass index
CI: Confidence interval
DPP-4: Dipeptidyl-peptidase-4
GLP-1: Glucagon-like Peptide 1
HbA_1c_: Hemoglobin A_1c_
IGF-1: Insulin-like growth factor-1
LKB1: Liver kinase B1
mTOR: mammalian target of rapamycin
OR: Odds ratio
RAGE: Receptor for AGE
RCT: Randomized Controlled Trial
ROS: Reactive oxygen species
SD: Standard deviation
SU: Sulfonylurea
T2DM: Type 2 diabetes mellitus.
